# Experimental Evidence of a Dirac Gap Opening in Carbon-Doped Topological Insulator Bi_2_Se_3_

**DOI:** 10.3390/nano16030205

**Published:** 2026-02-05

**Authors:** Qiya Liu, Xinsheng Yang, Min Zhang

**Affiliations:** 1 School of Physics and Astronomy, China West Normal University, Nanchong 637002, China; 2 College of Optoelectronic Technology, Chengdu University of Information Technology, Chengdu 610225, China; 3 Key Laboratory of Advanced Technology of Materials (Ministry of Education), Superconductor and New Energy R&D Center (SRDC), Southwest Jiaotong University, Chengdu 610031, China

**Keywords:** magnetic topological insulators, Bi_2_Se_3_, topological surface state, angle-resolved photoemission spectroscopy (ARPES), Shubnikov–de-Hass (SdH) oscillations, spin dynamic

## Abstract

Magnetic topological insulators (TIs) are promising candidates for realizing the quantum anomalous Hall effect (QAHE) and advancing the development of next-generation low-energy transistors and electronic devices. Doping Bi_2_Se_3_ with nano-carbon can introduce magnetic order and open the Dirac gap without introducing extrinsic magnetic impurities. In this work, the C_0.06_Bi_2_Se_3_ single crystal was prepared using the Bridgman method, and their electrical and magnetotransport properties were systematically investigated. Temperature-dependent resistivity and magnetoresistance measurements revealed a magnetic-field-induced metal–insulator-like transition near 152 K. Angle-resolved photoemission spectroscopy (ARPES) detected an energy gap of about 43 meV at the Dirac point, confirming that carbon doping modulates the surface state and opens the gap. Pronounced Shubnikov–de Haas oscillations indicate high carrier mobility in C_0.06_Bi_2_Se_3_. Furthermore, the temperature-dependent Kerr spectra shows that the spin relaxation behavior of C_0_._06_Bi_2_Se_3_ differs significantly from that of pure Bi_2_Se_3_; the relaxation process of spin electrons from the surface state (*τ*_s_) dominates the spin dynamics and exhibits distinct trends around 30 K and 150 K due to the interplay of the Dirac gap and impurity-induced states. These results demonstrate the potential of magnetic topological insulator C_0.06_Bi_2_Se_3_ for novel electronic and spintronic applications.

## 1. Introduction

Magnetic topological insulators (TIs) have attracted intensive research interest in the field of condensed matter physics for its magnetism, topology, unique electrical properties, and optical properties [1,2,3]. Bi_2_Se_3_ is a typical topological insulator; since the Fe substitution results in the opening of a gap found at the surface state by the angle-resolved photoemission spectroscopy (ARPES) experiments [4], it has been proven to be an effective way to obtain magnetic topological materials by doping topological insulators with transition group elements such as Cr and Fe [5]. Time-reversal symmetry breaking owing to magnetic field or magnetic impurities is expected to open an energy gap and destroy the topologically protected states. An energy gap at the Dirac point can cause many exotic phenomena, such as magnetic monopole [6], anomalous quantum Hall effect (AQHE) [7,8], and Kerr magneto-optical effects [9], which will further broaden the research and potential applications of topological insulators.

Additional ferromagnetic orders are introduced by doping of magnetic elements, and associated questions like unclear magnetic sources are anticipated. The mechanism of magnetism is complex and controversial. Mn-doped Bi_2_Se_3_ can exhibit a spin glass state, paramagnetic state and ferromagnetic state [10,11,12,13,14], and the ferromagnetic–paramagnetic transition occurs with the increase in Mn concentration [15]. Li [16] reported that the ferromagnetism in Fe-doped Bi_2_Se_3_ originates from the second phase rather than the intrinsic one. It has been reported that the reason why the Fe- and Ni-doped Bi_2_Se_3_ samples exhibit mixed magnetism such as diamagnetism, paramagnetism and ferromagnetism is that a small number of ferromagnetic compounds are formed on the surface of the samples [17,18,19].

On the other hand, some research groups have tried to introduce magnetic order by nonmagnetic doping. The 2p light elements (B, C, and N) doping at anion sites can produce magnetic moments and a gap opening at the Dirac point through the density functional theory [20,21,22]. The experiment shows clear hysteresis loops at 15 K, which suggests the existence of a ferromagnetic state in C_x_Bi_2_Se_3_ [23]. An obvious advantage of nonmagnetic element doping is that the magnetic clusters or secondary phases formed by the dopants do not contribute to magnetism. Nevertheless, there has not been enough transport and energy band experimental information to demonstrate the peculiar transport phenomena by nonmagnetic doping. Thus, we report on the transport properties of C_0.06_Bi_2_Se_3_ for understanding modulation electronic properties.

However, the electromagnetic transport properties of these samples cannot directly show the regulation of nonmagnetic elements on the electronic structure and surface state of Bi_2_Se_3_. In order to further study the influence of C element on Bi_2_Se_3_ electronic structure and Dirac energy gap, first-principles calculations and angle-resolved photoelectron spectroscopy (ARPES) have been used to study the band structure of single-crystal samples. Previously, first-principles calculations have shown that 2p light element C is the most effective dopant to induce the magnetic ground state of Bi_2_Se_3_. Chen et al. have performed ARPES to investigate the band structure of intrinsic, nonmagnetically doped, and magnetically doped Bi_2_Se_3_ [4]. But the band structure of C-doped Bi_2_Se_3_ has not been experimentally reported. Therefore, we used ARPES to distinguish the band structure of C_0.06_Bi_2_Se_3_ for understanding the regulatory effect of C impurity on the Fermi energy (E_F_) and surface state.

Notably, the surface states, which are protected by time-reversal symmetry, possess properties such as spin-momentum locking, backscattering protection [24,25] and high mobility [26]. The surface bands constituting a Dirac cone and the regulation of the Dirac gap of Bi_2_Se_3_ are hence crucial aspects that affect the potential applications of these materials in novel electronic and spintronic devices [27,28]. However, optical spin control of Dirac surface states (SS) remains one of the most challenging problems governing their potential applications [29,30]. A long spin lifetime ensures that information carried by spin is not lost during device operation. It is therefore important to examine the interaction of light with SS and understand the resulting charge carrier and spin dynamics [31,32,33]. However, a fast control of spins is a major quest in spintronic systems. Ultrafast optical pump–probe spectroscopy has been utilized to trigger and detect the spin dynamics of electrons in magnetic materials and multilayers [27,28,29,30,31,32,33,34]. In this paper, the ultrafast optical pump–probe spectroscopy is used to study the temperature dependence of the relaxation times of electron–electron scattering and electron–phonon scattering in C_0.06_Bi_2_Se_3_. These results will be helpful to explore the influence of impurities scattering on the electrical transport characteristics of C_0.06_Bi_2_Se_3_.

## 2. Experimental Details

**Figure 1:**
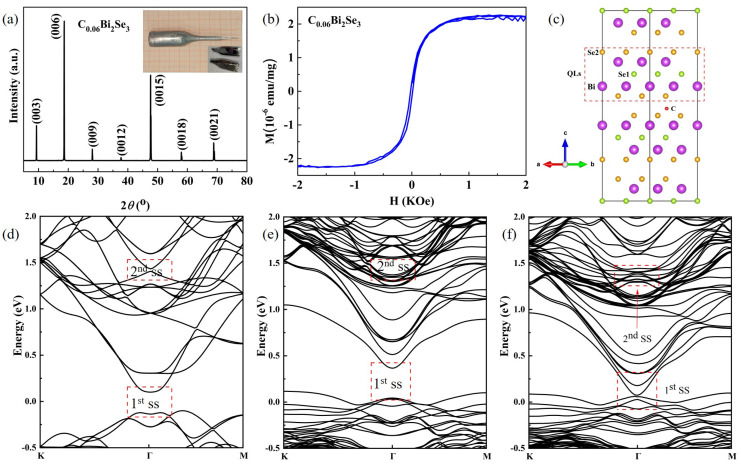
(**a**) X-ray diffraction patterns of C_0.06_Bi_2_Se_3_ crystal. Inset: a photograph of the as-grown crystal. (**b**) Hysteresis (M-H) curves of the C_0.06_Bi_2_Se_3_ crystal measured at 20 K, demonstrating ferromagnetism. (**c**) Schematic representation of the crystal structure of C_0_._06_Bi_2_Se_3_, illustrating the primary mechanism of carbon intercalation in the van der Waals gap between quintuple layers. (**d**) Band structure of pristine Bi_2_Se_3_ calculated with spin–orbit coupling (SOC). (**e**,**f**) Band structures of C_0_._06_Bi_2_Se_3_ calculated without (**e**) and with (**f**) SOC. The red dashed boxes highlight the regions of the topological surface states.

Single crystal C_0.06_Bi_2_Se_3_ was prepared by the modified Bridgman method using high-purity bismuth (99.999%), selenium (99.999%) and nano-carbon (99.99%) powder as starting materials (Aladdin, Shanghai, China). The quartz ampoule was placed in the upper (hotter) zone of the Bridgman furnace and heated to 1170 K to fully melt the constituents. During the growth process, the ampoule was continuously rotated at 45 rpm about its axis to enhance the radial uniformity of carbon distribution. It was then lowered into a cooler zone maintained at ~950 K at a rate of 1.6 mm/day. The as-grown single crystal measured approximately 87 mm in length (see inset of Figure 1a), displayed excellent cleavability, and was oriented with its trigonal axis (*c*-axis) perpendicular to the pulling direction—i.e., the (0001) crystal plane remained parallel to the ampoule axis.

The band structure of C_0.06_Bi_2_Se_3_ was determined using the first-principle calculations using the density functional theory (DFT) method set as implemented in the Vienna Ab initio Simulation Package [35,36]. The general gradient approximation (GGA) with Perdew–Burke–Ernzerhof (PBE) [37] functional is used to approximate the exchange–correlation functional. The Monkhorst–Pack k-mesh of 3 × 3 × 1 and cut-off energy of 500 eV are set. The vdW correction proposed by Grimme (DFT-D2) is used in the structure relaxation. The band structures are calculated with the spin–orbit coupling (SOC).

Angle-resolved photoemission spectroscopy (ARPES) was conducted at U9-CGM BL21B1 beamline at National Synchrotron Radiation Research Center in Hsinchu, Taiwan. The photoemission spectra were measured in a UHV chamber equipped with a hemispherical analyzer (Scienta R4000) with collecting angle ±15 deg. The polarization vector was invariably in the angular dispersive plane. The samples were cleaved in situ and measured at a base pressure 4.9 × 10^−11^ Torr. All spectra were recorded at 20 K and at 20 eV photon energy. The angular resolution was 0.2 deg; the energy resolution was about 9 meV.

All the measurements were performed on the new cleaved pristine surface. Resistivity and Hall effect measurements were performed in a quantum design physical property measurement system (PPMS, Quantum Design, USA).

Using femtosecond (fs) pump–femtosecond (fs) probe spectroscopy (Coherent, USA), the Δ*R*/*R*_0_ (t) measurements were performed using a commercial mode-locked Ti: sapphire laser system (~35 fs pulse duration, 800 nm center wavelength, and 80 MHz repetition rate). Both pump and probe laser beams were focused onto the sample with a spot diameter of ~30 μm^2^. The pump and probe beam fluence was 10.0 J/cm^2^ and 1.00 J/cm^2^, respectively.

## 3. Results and Discussion

### 3.1. Crystal Structure and Band Structure

Figure 1a shows the XRD pattern of the C_0.06_Bi_2_Se_3_ sample. The (0 0 *l*) family of diffraction peaks is observed with no apparent impurity phase, indicating that the sample is single-crystal and the quintuple layers (QLs) are stacked along the *c*-axis. A refinement of the lattice parameters reveals a slight contraction compared to pristine Bi_2_Se_3_ (see Supplementary Information), consistent with the incorporation of smaller carbon atoms. In contrast to the diamagnetic behavior of pure Bi_2_Se_3_, the M-H curves of C_0.06_Bi_2_Se_3_ exhibit clear ferromagnetism (FM) at low temperature, as shown in Figure 1b. This confirms that the C impurity induces a ferromagnetic state in Bi_2_Se_3_. To illustrate the primary doping mechanism under investigation, a structural model is presented in Figure 1c. In the QLs, C impurity is close to the Se1 position of the interlayer. Based on the previous research results, we found that the C impurity is doped into Bi_2_Se_3_; C mainly functions as intercalation for QLs as the doping mode at low concentrations. When the concentration exceeds 6%, some C atoms enter the QLs and the van der Waals gap QL-QL [21,22,23]. Therefore, in this paper, an independent structure for the C intercalation modes was designed for experimentation and calculation. This schematic highlights the intercalation of carbon into the van der Waals (vdW) gap between QLs, which is supported by first-principles calculations indicating it as the most stable configuration at low concentrations and the key mechanism for inducing magnetic moments and breaking time-reversal symmetry [20,22]. We note that the real material contains inherent defects such as Se vacancies, and the introduced C atoms may interact with this defective landscape. However, this simplified model focuses on the dominant effect of the C intercalant itself on the electronic and magnetic properties.

The band structure was calculated using first principles to further understand the influence of C impurity on the surface state of Bi_2_Se_3_. The results are shown in Figure 1d–f. Figure 1d displays the band structure of Bi_2_Se_3_ with SOC, and the positions marked by the red dotted boxes belong to the first surface states (1st SSs) and the second surface states (2nd SSs), respectively. Here, a Dirac cone can be observed with the energy band reversed at the Γ point; this result is similar to the calculation results of Wang et al. [21] and Xu et al. [38]. Figure 1e,f show the band structure of C-doped Bi_2_Se_3_ via the DFT. The calculation results of both schemes (without SOC and with SOC) show that the introduction of C impurity will enhance the surface state effect of Bi_2_Se_3_. Especially when SOC is considered in the calculation process, the surface state effect is enhanced more significantly. The calculation results of the paper are consistent with the results obtained by Zhang et al. for the topological surface states of Bi_2_C*_x_*Se_3_, implying that C impurity regulates the Bi_2_Se_3_ surface states via spin–orbit coupling and the spontaneous polarization of the C atom [22,23].

**Figure 2:**
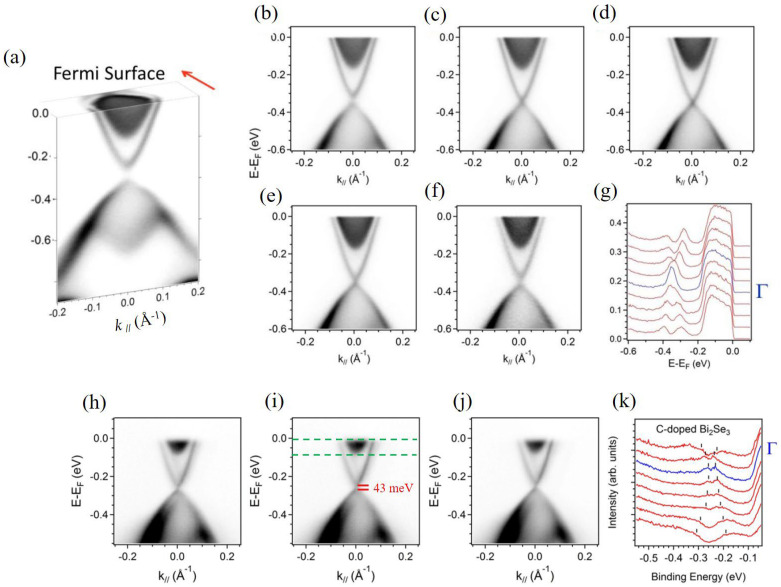
(**a**) Band-mapping result of various tilting angles between the surface normal and detector. (**b**–**f**) A series plot of band mapping of photoemission intensity conducted with 0.1 degree interval of tilting angles; (**g**) energy distribution curve at the Γ point for intrinsic Bi_2_Se_3_; (**h**–**j**) the band-mapping plots of photoemission intensity for C-doped Bi_2_Se_3_ with x = 0.06. Green lines mark the Fermi level and the bottom of the conduction band, *E* − *E_F_* = −80 meV. (**k**) EDC with measured cuts across the Γ point.

In Figure 2 all the samples were tested at 20K, and the typical band-mapping plots of ARPES spectra in intrinsic Bi_2_Se_3_ was displayed. A linear Dirac-cone-like dispersion relation can be clearly observed in Figure 2a. The topological surface state with the Dirac point located at 0.35 eV binding energy below the Fermi level is clearly observed in the plot, suggesting the n-type nature in highly crystalline Bi_2_Se_3_ sample, which can be seen in Figure 2a. ARPES results on intrinsic Bi_2_Se_3_ show the Fermi level 200 meV above the bottom of the conduction band.

As shown in Figure 2b–f, a series plot of band mapping of photoemission intensity is conducted by tilting the angle between the surface normal of cleaved crystal plane and detector with 0.1 degree accuracy, ensuring the measured cut can correctly pass through the Γ point. A linearly crossed conical band is observed near point Γ, while a parabolic band appears near the conduction band. The linear conical band represents the topological surface state, whereas the parabolic band corresponds to the bulk conduction band. By analyzing the fitting result of the energy distribution curve (EDC) at the Γ point, shown in Figure 2g, only a single peak at 0.35 eV indicates no gap observed in pure Bi_2_Se_3_. The same method was used to examine whether a gap opening exists in carbon-doped Bi_2_Se_3_ compounds.

Figure 2h–k display the band-mapping plots of photoemission intensity with measured cuts across the Γ point with varied carbon content in C_0.06_Bi_2_Se_3_ crystals. A small band-gap opening around the Dirac point was observed in C_0.06_Bi_2_Se_3_ crystals at 20 K.

In Figure 2k, a series of EDCs around the Γ point for C-doped Bi_2_Se_3_ samples are displayed; an existing band gap around the Dirac point can be identified clearly from the EDC at the Γ point and the fitting values of the band gap in the sample with x = 0.06 is 43 meV. The change in surface state and the electrons behaving like type-II Dirac fermions even without applying an external magnetic field in Figure 2h–j are mainly due to ferromagnetic ordering generated by the C dopants inside the material. The ARPES spectra reveals *E_F_* of around 80 meV above the bottom of the conduction band.

### 3.2. Electrical and Magnetic Transport Characteristics

**Figure 3:**
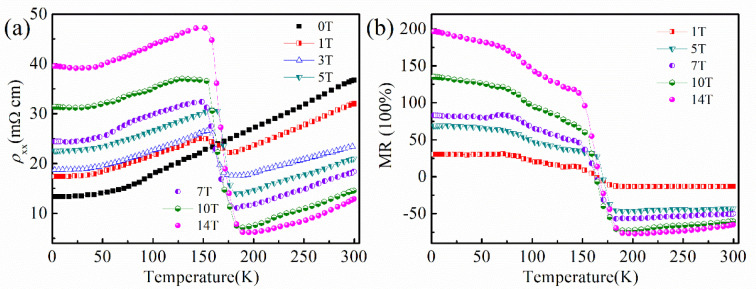
(**a**) Temperature dependence of the resistivity *ρ*_xx_ measured under different magnetic fields. (**b**) The MR with applied fields.

Figure 3a shows the temperature-dependent resistivity *ρ*_xx_ on a sample with a magnetic field applied perpendicular to the applied current in the *ac* plane. At zero field, *ρ*_xx_ exhibits metallic behavior. With *ρ*_xx_ (300 K) = 36.77mΩcm and *ρ*_xx_ (2 K) = 13.35mΩcm, the residual resistance ratio (RRR), R (300 K)/R (2 K) is about 2.75. The resistivity of the sample increases slightly below 30 K, and this trend still exists under the action of a magnetic field. Here, the resistivity is contributed by the surface Dirac cone and bulk conduction. At low temperatures (<30 K), the bulk bands are gapped, leading to surface Dirac cone conduction being the dominant conduction mechanism. As the temperature rises, the gap decreases, and the bulk conduction becomes dominant.

When a magnetic field is applied along the *b* axis, the temperature-dependent resistivity *ρ*_xx_ in the ac plane increases dramatically in low temperatures. Near 152 K, a clear transition occurs where resistivity rises with decreasing temperature, leading to insulation-like behavior for fields above 1 T. This magnetic field-induced metal–insulator-like transition, commonly observed in topological semimetals [39,40], can be explained by the following mechanism in our C-doped Bi_2_Se_3_, which behaves as a topological semiconductor. Analogous to the behavior in compensated semimetals like WTe_2_ [39], where extreme magnetoresistance arises from near-perfect electron–hole compensation and high carrier mobility, the applied magnetic field here may enhance scattering or modify the relative contributions of surface and bulk conductance. In particular, the magnetic field likely suppresses bulk conductivity—already reduced by the C-induced gap—while the surface channels, though present, are insufficient to maintain metallic conduction. This leads to an overall insulation-like temperature dependence in *ρ*_xx_(B, T) above a critical field, as the system enters a regime where conduction is dominated by a low-density, high-mobility channel that is highly sensitive to magnetic confinement and scattering. Therefore, the transition reflects a field-tuned crossover from bulk-dominated metallic transport to a state where the remaining conductive paths are strongly localized or magnetically suppressed.

In order to phenomenally describe the metal–insulator phase transition caused by the magnetic field, we use the Arrhenius equation ($\rho \propto exp[-E_{g}/(k_{B}T)])$, where *ρ*, *E*_g_, *k_B_* and T are resistivity, energy gap, Boltzmann constant and absolute temperature, respectively. Data fitting and analysis gives the activation energy *E*_g_ in Table 1. It can be calculated that the *E*_g_ = 97.2 meV for 7 T, which is similar to the 97 meV for 7 nm Bi_2_Se_3_ film on Si [41]. It indicates that there is a shallow impurity band from the Se vacancies and C-doping effect located about 97.2 meV lower than the bulk conduction band.

Figure 3b shows the magnetoresistance (MR) of the C_0.06_Bi_2_Se_3_ crystal versus temperature (MR-T), where MR is calculated as the ratio of the change in resistivity due to the applied magnetic field (H); MR = [(*ρ*_xx_(B) − *ρ*_xx_(0))/*ρ*_xx_(0)] × 100%. Obviously, MR increases as field increases. The MR of C_0.06_Bi_2_Se_3_ crystal is as large as 200% measured at 2 K and 14 T magnetic field. With decreasing temperature, MR first decreases, reaching a minimum around 152 K, and then increases. Simultaneous crossovers in *ρ*-T and MR-T characteristics at around 152 K are observed. This occurrence indicates that the SS contributions are significantly improved upon doping with C concentrations, which in consistent with the results of our later study on Shubnikov-de-Hass (SdH) oscillations in this paper.

**Figure 4:**
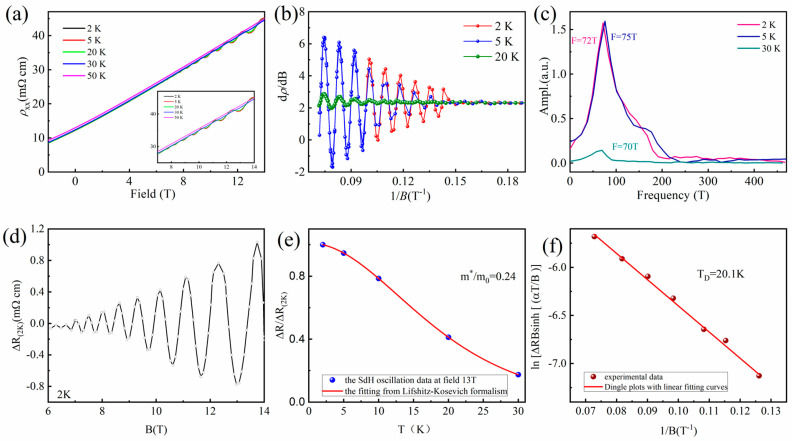
(**a**) Temperature-dependent resistivity of C_0.06_Bi_2_Se_3_ crystal under different applied magnetic fields. Inset: SdH oscillation appears above 6T. (**b**) The d*ρ*_xx_/dB-1/B curves of C_0.06_Bi_2_Se_3_ crystals sample. (**c**) The corresponding Fourier transform result is plotted, giving the SdH oscillation frequencies. (**d**) The oscillatory component ΔR as a function of B at 2 K. (**e**) SdH oscillation amplitudes at B = 13 T. The solid curves are fitted to the Lifshitz–Kosevich formula from 2 to 50 K. The corresponding cyclotron masses are determined to be m* = 0.24m_0_. (**f**) ln [ΔR*B*sinh[(*α*T/B)]] plotted as a function of 1/B. Dingle plots with linear fitting curves, which provide the Dingle temperatures T_D_ = 20.1 K.

Hall measurements clearly verify that the sample is n-type, with a bulk carrier density of 1.1 × 10^19^ cm^−3^. There is clear SdH oscillation at the low temperatures and the high magnetic fields above ∼6T, as shown in Figure 4a, which indicates the high mobility in C_0.06_Bi_2_Se_3_ [39,42]. The amplitude of the SdH oscillations decreases with increasing T, and the oscillations are not observed for T > 50 K. To remove the smooth background and analyze the SdH oscillations, the d*ρ*_xx_/dB-1/B curves in the high fields are plotted in Figure 4b. As can be seen, the d*ρ*_xx_/dB values show periodic oscillations as the function of 1/B. By taking the Fourier transformation of the oscillations, the oscillation frequencies are obtained as F = 72 T, 75 T and 70 T for 2 K, 5 K and 20 K, respectively, as shown in Figure 4c. We fitted the data of 2 K by using the Onsager relation F = ℏA/2πe [43], where A =$\;\pi k_{F}^{2}$ is the cross-sectional area of the Fermi surface with the 2D relation $n_{s}=\frac{k_{F}^{2}}{4\pi }$. The corresponding wave vector *k*_F_ is 0.0477Å. The sheet carrier concentration *n*_s_ is 1.818 × 10^12^ cm^−2^. Only a single oscillation frequency is observed, implying that only the top surface contributes to the observed SdH oscillations [21]. The SdH frequency F is 70 T for 20 K corresponding to a Fermi energy *E_F_* of 79 meV, consistent with the ARPES-observed *E_F_* of approximately 80 meV.

Figure 4d presents the Shubnikov–de Haas (SdH) oscillation signal, denoted as ΔR_(2K)_, which is obtained by subtracting a smoothly varying polynomial background, ρ_xx_(background), from the measured magnetoresistance *ρ*_xx_(2 K) at 2 K in the high-field regime (i.e., ΔR(2 K) = *ρ*_xx_(2 K) − *ρ*_xx_(background)). This process isolates the quantum oscillatory component for detailed analysis.

To quantify the temperature dependence of the oscillation amplitude, the relative amplitude ΔR/ΔR_(2K)_ is analyzed. Here, ΔR represents the oscillatory component (background-subtracted) at a given temperature, and ΔR_(2K)_ serves as the normalization reference at the base temperature of 2 K. The relative oscillation amplitude, defined as ΔR/ΔR_(2K)_, is plotted as a function of temperature, as shown in Figure 4e. The temperature dependence of the SdH oscillation amplitude was analyzed at a fixed magnetic field of B = 13 T. The solid line is a fit to the standard Lifshitz–Kosevich (LK) formula governing the thermal damping of SdH oscillations [42]: $\frac{A(T)}{A(T_{0})}=\frac{Tsinh(T_{0}x)}{T_{0}\cdot sinh(Tx)}$, where T_0_ = 2 K, *x* =$\;\frac{\beta m^{\prime }}{B}$, $m^{\prime }=m^{*}/m_{0}$, and $\beta =\frac{2\pi^{2}k_{B}m_{0}}{\hbar e}=14.7$. m* is the cyclotron effective mass. Figure 4e shows such a fitting with m* = 0.24m_0_ (m_0_ = 9.1 × 10^−31^ kg is the electron mass), which is consistent with previous measurements [44,45].

Further analysis of the scattering time is performed via the Dingle plot. The Dingle temperature T_D_ = ℏ/2π*τk*_B_ is the equivalent temperature increment which yields the field dependence of the oscillation, where *τ* is the quantum lifetime of carriers due to scattering, and *k*_B_ is Boltzmann’s constant. A higher T_D_ implies stronger impurity scattering. T_D_ is found to be 20.1 K from the slope of the linear fit with ln [ΔR*B*sinh [(*α*T/*B*)]] and $\alpha =\frac{2\pi^{2}m^{*}k_{B}}{\hbar e}\;$ in Figure 4f. The carrier lifetime *τ =*$\;\frac{\hbar }{2\pi {T_{D}k}_{B}}$ was extracted 8.3 × 10^−14^ s from T_D_. According to the relation $\mu_{s}=\frac{e\tau }{m^{*}}$ the quantum effective mobility of electrons is 608 cm^−2^ V^−1^ s^−1^. These results are consistent with previous reports in order of magnitude [46,47].

### 3.3. Time-Resolved Kerr Spectrum and Spin Dynamic

**Figure 5:**
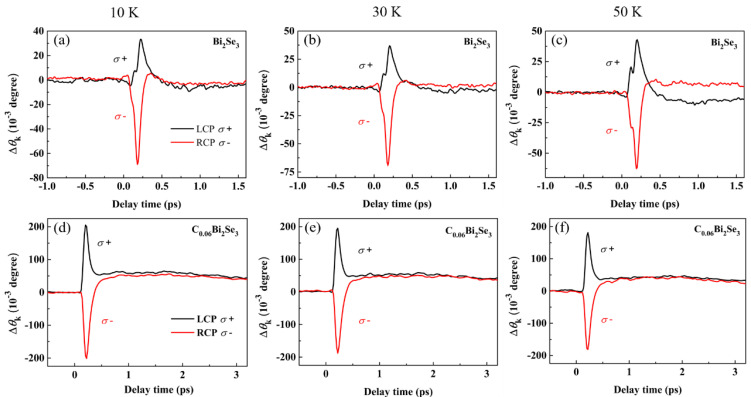
Time-resolved Kerr rotation Δ*θ*_k_ induced by left (*σ*^+^) and right (*σ*^−^) circularly polarized light at 10 K, 30 K, and 50 K. (**a**–**c**) Bi_2_Se_3_; (**d**–**f**) C_0.06_Bi_2_Se_3_.

The time-resolved Δ*θ*_k_(*t*) signals of the Bi_2_C*_x_*Se_3_ (x = 0, 0.06) crystals were acquired using the fs pump–probe technique at temperatures of 10 K, 30 K, and 50 K, as illustrated in Figure 5. The Δ*θ*_k_ signals were induced by circularly polarized light injected in the samples: left-handed circular polarization (denoted as *σ*^+^), and right-handed circular polarization (denoted as *σ*^−^). Theoretically [48] and experimentally [49], the circularly polarized light is used to excite only one branch of the spin-momentum-locked surface states in TIs. According to the spin selection rules, the net spin density is calculated based on the circularly polarized light in opaque materials, which includes the direct optical transitions between different energy levels and accompanying angular momentum transfer [50]. Therefore, the Δ*θ*_k_ signals are generated owing to the transition of the spin electrons in the surface state when the sample is excited by circularly polarized light. From Figure 5a–c, we observe that the evolution of σ^+^ and σ^−^ signals is different with time in pure Bi_2_Se_3_ crystals. With increasing temperature, the σ^+^ signals increase and the σ^−^ signals decrease. A similar phenomenon has been reported by Wang et al. [33] and Hsieh et al. [51]. The reason for this phenomenon may be the band-gap shrinkage with increasing T owing to electron–phonon interactions [52]. However, these phenomena are not observed in C_0.06_Bi_2_Se_3_. The Δ*θ*_k_(*t*) signals of σ^+^ and σ^−^ remain nearly constant at different temperatures, as shown in Figure 5d–f. These results indicate that the spin polarization of C impurity regulated the surface state and band structure of Bi_2_Se_3_ and enhanced the surface state in polarization. This is consistent with the results obtained from the band structure shown in Figure 1d–f.

**Figure 6:**
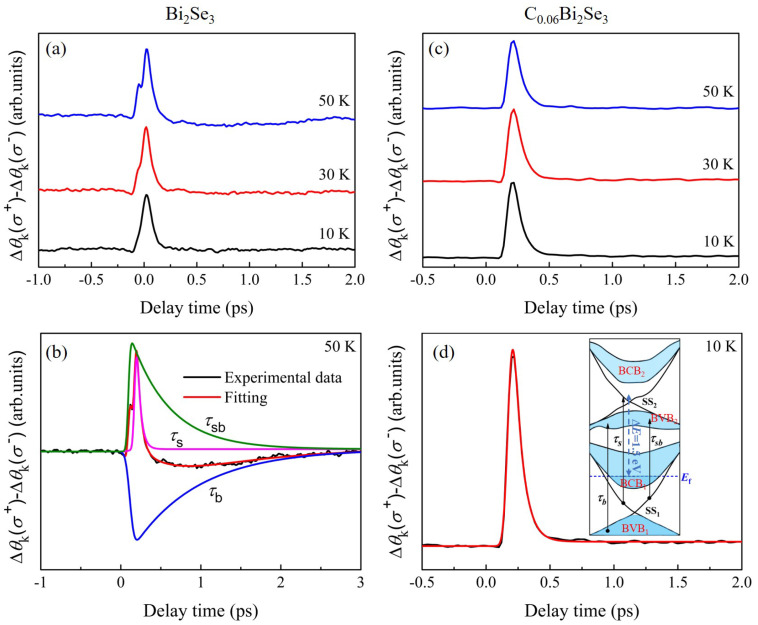
(**a**) [Δ*θ*_k_ (*σ*^+^)-Δ*θ*_k_ (*σ*^−^)] of Bi_2_Se_3,_ where [Δ*θ*_k_ (*σ*^+^)-Δ*θ*_k_ (*σ*^−^)] ≈ 2Δ*θ*_k_ (*σ*^+^). (**b**) Exponential decay fittings (red lines) for experimental Δ*θ*_k_ at 50 K (black lines). Green, pink, and blue lines describe three distinct dynamical processes characterized by *τ*_sb_, *τ*_s_, and *τ*_b_, respectively. (**c**) [Δ*θ*_k_ (*σ*^+^)-Δ*θ*_k_ (*σ*^−^)] of C_0.06_Bi_2_Se_3_. (**d**) Exponential decay fittings (red lines describe the dynamical processes characterized by *τ*_s_) for experimental Δ*θ*_k_ at 10 K (black lines). Inset: Schematic representation of the three distinct dynamical processes *τ*_sb_, *τ*_s_, and *τ*_b_ in Bi_2_Se_3_.

To further understand the effect of the C impurity on the spin dynamics in Bi_2_Se_3_, we investigated the [Δ*θ*_k_ (*σ*^+^)-Δ*θ*_k_ (*σ*^−^)] of Bi_2_C*_x_*Se_3_. Figure 6a,c show the [Δ*θ*_k_ (*σ*_+_)-Δ*θ*_k_ (*σ*^−^)] of Bi_2_Se_3_ and Bi_2_C*_x_*Se_3_. Compared with the [Δ*θ*_k_ (*σ*^+^)-Δ*θ*_k_ (*σ*^−^)] of Bi_2_C*_x_*Se_3_ crystals with different temperatures, a small peak is shown on the left side in [Δ*θ*_k_ (*σ*^+^)-Δ*θ*_k_ (*σ*^−^)] of Bi_2_Se_3_, which becomes highly evident with the increase in *T*. Using a multiple exponential decay relaxation model, we fit the [Δ*θ*_k_ (*σ*^+^)-Δ*θ*_k_ (*σ*^−^)] of Bi_2_Se_3_ at 50 K. The fitting equation is expressed as follows:

(1) $$\Delta \theta_{k}\left(t\right)=A_{s}\exp\left(-\frac{t-t_{0}}{\tau_{s}}\right)+A_{sb}\exp\left(-\frac{t-t_{0}}{\tau_{sb}}\right)+A_{b}\exp\left(-\frac{t-t_{0}}{\tau_{b}}\right)+C$$

where *A_s_*, *A_sb_*, and *A_b_*, as well as *τ_s_*, *τ_sb_*, and *τ_b_* are the amplitude and relaxation times of the decay of spin carriers, respectively, via different relaxation mechanisms. C is a constant.

In Figure 6b, three dynamic processes contribute to Δ*θ*_k_(*t*) signals of Bi_2_Se_3_, and the fitting results are listed in Table 2. According to Wang et al., the three relaxation processes (*τ*_s_, *τ_b_* and *τ_sb_*) are attributed to the spin relaxation of electrons from the transition of SS_1_ and BVB_1_ to SS_2_ and BVB_2_, via SS_1_ → SS_2_ (*τ_s_*), BVB_1_ → BVB_2_ (*τ_b_*), and SS_1_ → BVB_2_ (*τ_sb_*), respectively [33]. And a diagram of the various processes is shown in the illustration in the inset of Figure 6d.

In Bi_2_Se_3_, the phonons are “frozen” at low temperatures, the bulk bands are gapped, and the electromagnetic transport behavior is dominated by the surface states when *T* < 30 K. As the temperature increases, the phonons of bulk state become more active, and phonon scattering dominates the electromagnetic properties [53]. Due to the influence of the bulk state A_1g_^1^ phonon scattering, the *τ_sb_* and *τ_b_* change with temperature [33,54]. Notably, the process characterized by *τ*_s_ is attributed to the relaxation of the spin-polarized electrons in SS_2_ via SS_1_ → SS_2_, and the spin-polarized electrons can be generated by SS-to-SS interband transitions with particular spin selection rules using circularly polarized light. According to the theoretical works of Lu et al. [55] and Hosur et al. [48], the corresponding Δ*θ*_k_(*t*) signal should vanish with decreasing temperature, in good consistency with our experimental observations in Figure 5a and the decrease of *τ_sb_* with decreasing temperature [see Table 2]. This result is consistent with that reported by Wang et al. [33] determined based on the spin-scattering mechanism of TSS. In addition, the surface state defects caused by Se vacancy in Bi_2_Se_3_ [56] may cause *τ*_s_ to change with temperature.

For further discussion *τ*_s_, the [Δ*θ*_k_ (*σ*^+^)-Δ*θ*_k_ (*σ*^−^)] of C_0.06_Bi_2_Se_3_ have been fitted by a single exponential model, as shown in Figure 6d, and the results are listed in Table 2. We observed that the *τ_s_* of samples remain nearly constant for temperatures ranging from 10 K to 50 K. According to Xin et al., C impurity introduces magnetism in Bi_2_Se_3_ through spontaneous polarization and enhances the surface state [22]. Therefore, we speculate that the C impurity enhances the effect of the surface state due to its spin polarization, resulting in a stable spin relaxation time *τ_s_*. Simultaneously, the spin relaxation (*τ_s_*) is closely related to spin interactions [57,58]. From Figure 1e, C impurity regulates the Bi_2_Se_3_ surface states via the various spin interactions. The results suggested that the surface state of Bi_2_Se_3_ is enhanced by the C impurity, and *τ_s_* is increased and stable due to the spin interaction. This scenario is also consistent with the theoretical works of Elliott and Yafet [57,58]. Their reports have pointed out that

(2) $$\tau_{s}^{-1}={\left(L_{eff}/\Delta E_{eff}\right)}^{2}\tau^{-1}\propto \tau^{-1}$$

Here, *L_eff_* is the average intensity of the momentum space of the SOC, Δ*E_eff_* is the width of the average energy gap, and *τ*^−1^ is the relaxation time of the electron’s momentum. When Δ*E_eff_t* ≫ 1, *τ_s_*^−1^ is proportional to *τ*^−1^, and its proportional coefficient (*L_eff_*/Δ*E_eff_*) will be affected by impurities, interfaces, phonon and other factors. In this work, *L_eff_*/Δ*E_eff_* is affected by C impurity, and *τ_s_*^−1^ of C_0.06_Bi_2_Se_3_ is different from Bi_2_Se_3_. These results also show the agreement between the experiment and the calculation.

**Figure 7:**
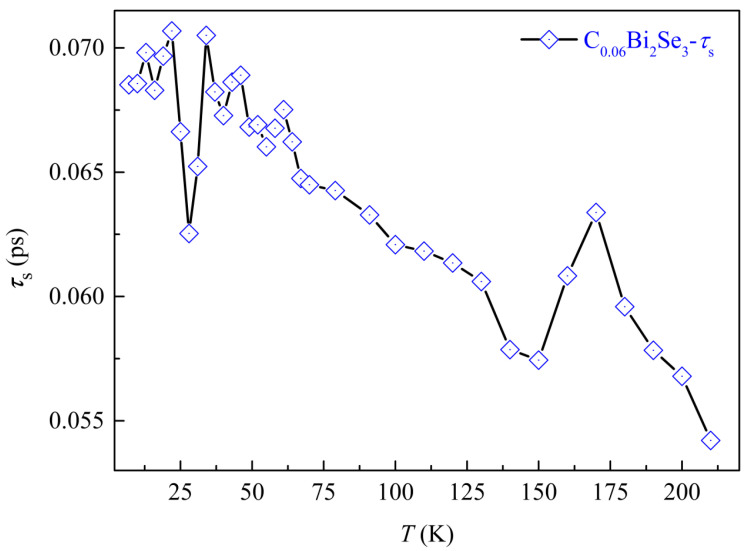
The temperature dependences of *τ*_s_ for C_0.06_Bi_2_Se_3_.

The temperature dependences of *τ*_s_ for C_0.06_Bi_2_Se_3_ shown in Figure 7. At low temperature (*T* ≤ 30 K), *τ_s_* increases with rising temperature, which may be related to the opening Dirac energy gap. The ARPES spectra shows that the Dirac point gap is about 43 meV; it may cause the surface state electron relaxation channel to be closed and *τ_s_* to become longer. But near the freezing temperature, the temperature dependence of *τ_s_* reverses, which may be related to the sudden enhancement of bulk state phonon scattering. This behavior is similar to the metal–insulator transition of Bi_2_Se_3_. Meanwhile, at this temperature, the gaps caused by impurities close again, providing a new channel for the relaxation of surface spin carriers. Then, with the increase in temperature and gap closure, the scattering effect of the bulk phonon activity becomes stronger, and as the relaxation channels for spin carriers continue to increase, *τ_s_* becomes shorter. However, the change in *τ_s_* reverses again when the temperature is near 150 K, which is consistent with the metal-insulation transition temperature with different magnetic fields. According to the results in Table 1, the C impurity leads to a large activation energy *E*_g_ at the temperature, which suppresses the relaxation process of the surface state spin electrons. The appearance of *E*_g_ indicates the presence of a gap caused by Se vacancies and C impurity below the conduction band. This gap will reduce the relaxation channels for spin carriers and increase their relaxation time. However, as the temperature continues to rise, the relaxation rates of each carrier increase continuously, and the *τ_s_* shortens.

In addition, we also observed that the *τ_sb_* and *τ_b_* hardly contribute to the Δ*θ*_k_(*t*) of C_0.06_Bi_2_Se_3_. This is because the C impurities in the Se vacancies of Bi_2_Se_3_ crystals, surface states, and band structures are adjusted according to the spin–orbit interaction in the material, which enhances the surface state of the materials. Moreover, the net spin relaxation signals from the surface state are enhanced, which causes the weaker net spin relaxation signals from the bulk state to be masked in the material.

## 4. Summary

In this study, C_0_._06_Bi_2_Se_3_ topological insulator single crystals were successfully synthesized via a modified Bridgman method. Through a combination of first-principles calculations, ARPES, electrical transport, and ultrafast spectroscopy, we provide the first experimental evidence that carbon doping opens a gap at the Dirac point in Bi_2_Se_3_. Resistivity measurements show that a magnetic-field-induced metal–insulator-like transition has been observed. Hall measurements clearly display SdH oscillation at the low temperatures and the high magnetic fields above ~6 T, with fitting data SdH frequency F = 70 T and Fermi energy *E*_F_ = 79 meV for 20 K. ARPES have provided evidence for the presence of a 43 meV gap around the Dirac point at 20 K. Ultrafast pump–probe spectroscopy further demonstrates that carbon doping markedly modifies the spin dynamics of the surface state, leading to a temperature dependence of the spin relaxation time *τ*_s_ that differs from that of the intrinsic sample, reflecting the competing effects of the Dirac gap and impurity states on spin scattering. This work not only offers a viable route to fabricating magnetic topological insulators via nonmagnetic doping but also lays an experimental foundation for future studies on the quantum anomalous Hall effect and magnetoelectric coupling devices.

## Figures and Tables

**Table 1:** The metal-insulation transition temperature *T*_m_ and extracted thermal active gaps *E_g_* at 6 T, 9 T for C_0.06_Bi_2_Se_3_.

B	*T_m_*	*E*_g_
1 T	153 K	80.3 meV
3 T	151 K	85.5 meV
5 T	152 K	94.6 meV
7 T	153 K	97.2 meV
10 T	152 K	90.5 meV
14 T	155 K	107.7 meV

**Table 2:** The relaxation time parameters of the different processes for the Bi_2_C*_x_*Se_3_ crystals.

	10 K	30 K	50 K
Bi_2_Se_3_-*τ_s_*	~37 fs	~68 fs	~46 fs
Bi_2_Se_3_-*τ_sb_*	~361 fs	~434 fs	~485 fs
Bi_2_Se_3_-*τ_b_*	~760 fs	~1064 fs	~818 fs
C_0.06_Bi_2_Se_3_-*τ_s_*	~67 fs	~68 fs	~68 fs

## Data Availability

The original contributions presented in this study are included in the article/Supplementary Materials. Further inquiries can be directed to the corresponding authors.

## Supplementary Materials

The following supporting information can be downloaded at: https://www.mdpi.com/article/10.3390/nano16030205/s1, Figure S1: (a) X-ray diffraction patterns of C_x_Bi_2_Se_3−x_ with different C concentration (x = 0.0, 0.04, 0.06, 0.1 and 0.15). (b) The relationship between lattice parameters and the C doping content. FESEM images for C_x_Bi_2_Se_3−x_ with different C concentration: (c) x = 0 and (d) x = 0.06.; Table S1: EDX analysis results for the C_x_Bi_2_Se_2_ series.
