# Supplementary material for: Experimental Evidence of a Dirac Gap Opening in Carbon-Doped Topological Insulator Bi2Se3
Source: Nanomaterials (Basel). 2026 Feb 5;16(3):205. doi: 10.3390/nano16030205 (PMC12899001; doi:10.3390/nano16030205)
Supplement: Supplementary file 1 [file nanomaterials-16-00205-s001.zip › nanomaterials-4068841-supplementary.pdf]

Supplementary Note: Preliminary Study on C-Doped Bi_2Se_3 Series

1. Sample Preparation and Structural Characterization

Prior to the detailed investigation presented in the main text, a series of C-doped samples with nominal compositions of $C_xBi_2Se_3$ (x = 0, 0.04, 0.06, 0.1, 0.15) were synthesized to preliminarily assess the doping effect on crystal structure and morphology.

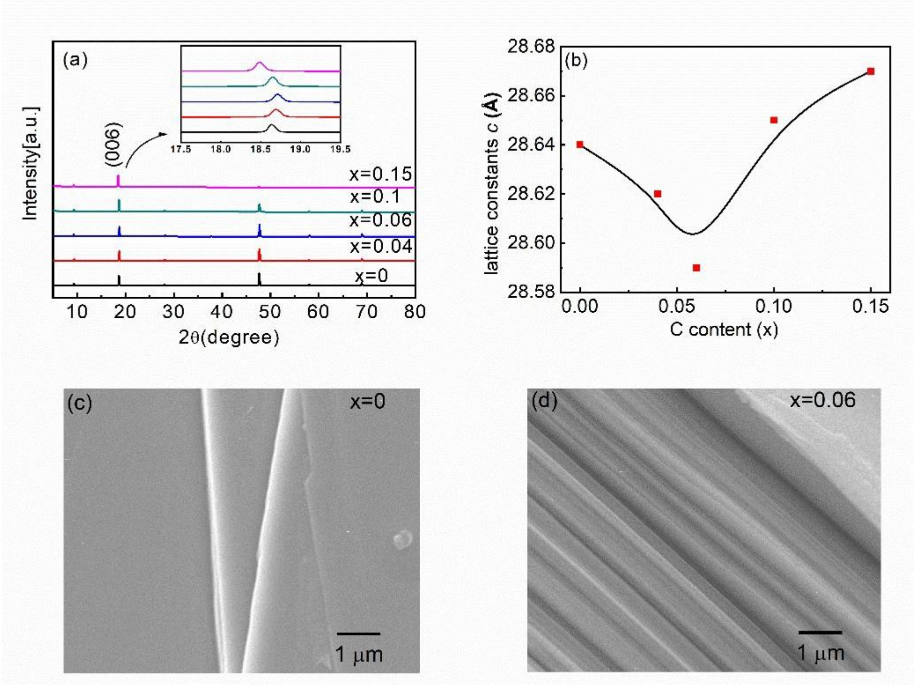

Fig. S1 (a) X-ray diffraction patterns of $C_xBi_2Se_{3-x}$ with different C concentrations (x=0.0，0.04，0.06，0.1 and 0.15). (b) The relationship between lattice parameters and the C doping content. FESEM images for $C_xBi_2Se_{3-x}$ with different C concentrations: (c) x = 0 and (d) x = 0.06.

X-ray diffraction (XRD) patterns of the series are shown in Supplementary Fig. S1a. All major diffraction peaks can be indexed to the rhombohedral phase of Bi_2Se_3 (space group R3m), and no secondary phases are detected within the sensitivity of XRD. The sharp diffraction peaks indicate high crystallinity. The full width at half maximum (FWHM) of the characteristic peaks is below 0.02° for samples with $x \leq$ 0.06 and remains under 0.03° for x = 0.1, confirming good crystal quality across the doping range.

A detailed analysis of the (0 0 6) peak position reveals a non-monotonic shift with increasing C content (Supplementary Fig. S1b). The peak first shifts to a higher angle for x = 0.04 and 0.06, suggesting a contraction of the lattice parameter *c*. For $x \geq 0.1$, the trend reverses, indicating lattice expansion. This complex behavior hints at a possible evolution of the carbon incorporation mechanism with doping concentration.

2. Morphological and Compositional Analysis

Field-emission scanning electron microscopy (FESEM) images confirm the layered structure of both undoped and C-doped crystals (Supplementary Fig. S1c, d). The surfaces appear smooth and cleavable. However, a practical observation is that the mechanical cleaving becomes progressively more difficult as the carbon content increases, implying a modification of the interlayer bonding.

Energy-dispersive X-ray spectroscopy (EDX) was employed to determine the actual chemical composition (Supplementary Table S1). The results confirm the presence of Bi, Se, and C in all doped samples. The measured carbon content consistently deviates from the nominal value, indicating a complex incorporation process. The Bi:Se ratio also shows variations, suggesting that carbon doping may be accompanied by the compensation or creation of native point defects (e.g., Se vacancies).

Supplementary Table S1. EDX analysis results for the $C_xBi_2Se_3$ series.

sample	Nominal component (at.%)			EDX test results (at.%)		
	C	Bi	Se	C	Bi	Se
0.0	0	40	60	0	40.02	59.98
0.04	0.8	40	59.2	0.6	40.51	58.89
0.06	1.2	40	58.8	1.6	40.98	57.42
0.1	2	40	58	2.43	38.62	58.95
0.15	3	40	57	3.17	39.5	57.33

3. Discussion: Mechanism of Carbon Doping

The combined structural, morphological, and compositional data suggest a dual incorporation mechanism for carbon in Bi_2Se_3, consistent with previous theoretical reports [20, 22].

Low doping levels ($x \leq 0.06$): The initial lattice contraction can be attributed to the predominant intercalation of smaller carbon atoms (atomic radius ~0.86 Å) into the van der Waals (vdW) gap between the quintuple layers (QLs). This intercalation may also partially passivate native Se vacancies (atomic radius ~1.60 Å), leading to a more compact local structure. The retained, though slightly reduced, cleavability supports the presence of C in the vdW gap, which modifies but does not completely replace the interlayer bonding.

Higher doping levels ($x \geq 0.1$): The subsequent lattice expansion and increased cleaving difficulty suggest a growing contribution from substitutional doping, where carbon atoms replace selenium within the QLs. The formation of stronger Bi-C covalent bonds in place of Bi-Se bonds can distort the local lattice and effectively increase the interlayer spacing. The significant deviation in EDX-measured composition from nominal values further supports a complex, non-stoichiometric incorporation process likely involving both interstitial (vdW) and substitutional sites, as well as related defect complexes.

These preliminary findings informed the selection of the $x = 0.06$ composition for the main study. At this concentration, the system is expected to be in a regime where intercalation is dominant, allowing us to isolate and investigate the effects of vdW-gap-doped carbon on the topological electronic and magnetic properties of Bi_2Se_3, as presented in the main manuscript.
